# MS4A6A genotypes are associated with the atrophy rates of Alzheimer's disease related brain structures

**DOI:** 10.18632/oncotarget.9563

**Published:** 2016-05-23

**Authors:** Jing Ma, Wei Zhang, Lin Tan, Hui-Fu Wang, Yu Wan, Fu-Rong Sun, Chen-Chen Tan, Jin-Tai Yu, Lan Tan, Alzheimer's Disease Neuroimaging Initiative

**Affiliations:** ^1^ Department of Neurology, Qingdao Municipal Hospital, School of Medicine, Qingdao University, Qingdao, China; ^2^ College of Medicine and Pharmaceutics, Ocean University of China, Qingdao, China

**Keywords:** Alzheimer's disease, MS4A6A, polymorphisms, phenotypes, brain structure, Gerotarget

## Abstract

Membrane-spanning 4-domains, subfamily A, member 6A (*MS4A6A*) has been identified as susceptibility loci of Alzheimer's disease (AD) by several recent genome-wide association studies (GWAS), whereas little is known about the potential roles of these variants in the brain structure and function of AD. In this study, we included a total of 812 individuals from the Alzheimer's disease Neuroimaging Initiative (ADNI) database. Using multiple linear regression models, we found *MS4A6A* genotypes were strongly related to atrophy rate of left middle temporal (rs610932: Pc = 0.017, rs7232: Pc = 0.022), precuneus (rs610932: Pc = 0.015) and entorhinal (rs610932, Pc = 0.022) on MRI in the entire group. In the subgroup analysis, *MS4A6A* SNPs were significantly accerlated the percentage of volume loss of middle temporal, precuneus and entorhinal, especially in the MCI subgroup. These findings reveal that *MS4A6A* genotypes affect AD specific brain structures which supported the possible role of *MS4A6A* polymorphisms in influencing AD-related neuroimaging phenotypes.

## INTRODUCTION

As the most common neurodegenerative disorder in the elderly, Alzheimer's disease (AD) is becoming a major challenge to the global health care system with the progressive increase burden on caregivers and society in most countries [1, 2]. Cognitive screening and detailed neuropsychological assessment still were the foundation of the diagnosis for AD dementia, but by the time the patient was diagnosed, the disease had been progressing for many years [3]. Recently, the latest guidelines on diagnosis of AD suggested that histological pathology (such as amyloid plaques, neurofibrillary tangles and so on) should be detected by specific abnormality on various biomarkers [4]. To date, multiple neuroimaging measures, including position emission tomography (PET), structural magnetic resonance imaging (MRI) and Pittsburgh Compound B position emission tomography (PiB-PET), had been used to detect the presence or absence of AD pathology [5-8], on account of they may infer the clinical progression in normal aging and MCI. Adult twin-studies showed these measures appeared to be explained by genetic factors with high heritability [9]. In addition, there is convincing evidence that these neuroimaging traits also be affected by genetic risk factors for AD which further confirm the important function for these genetic factors and suggest possible mechanisms through that they might be playing for AD.

Numbers of genome-wide association study (GWAS)-validated or GWAS-promising candidate loci were investigated and had been substantiated strongly related to neuroimaging or metabolic biomarkers in the AD process [10-14]. Membrane-spanning 4-domains, subfamily A, member 6A (*MS4A6A*), located in chromosome 11q12.1, has been identified as one of the most significantly associated risk locus with AD in serial recent large GWAS [15-24]. Although lots researchers found high levels of MS4A6A were associated with increased risk of AD, and in relation to AD-related neurofibrillary pathology and tau phosphorylation *in vitro* [25-28], whether the loci associate with the neuroimaging or metabolic biomarkers are still unclear.

In this article, we defined many brain regions as regions of interest (ROIs), including the main pathological change regions of AD and the atrophy of some regions in AD has been previously confirmed *via* MRI studies [29-33]. Then, we genotyped multi-loci in *MS4A6A* and searched the involvement of MS4A6A in the onset and progression of AD by investigating the influence of *MS4A6A* polymorphism on brain structure and function in the participants from the Alzheimer's Disease Neuroimaging Initiative (ADNI) dataset.

## RESULTS

### Characteristics of included subjects

Demographic features, cognitive status, number of *APOE* ε4 allele and neuroimaging phenotypes of AD, MCI and control subjects were summarized in Table 1. Finally, we recruited 281 cognitively normal (74.51±5.56 years), 483 MCI (72.28±7.45 years) and 48 AD patients (75.51±9.23 years) in our study. As we had expected, the frequency for the ε4 allele within ApoE gene was significantly higher in AD group than CN group. Based on various neuropsychological scales (including CDRSB, ADAS11, ADAS13, MMSE, RAVLT, ADAS-cog, etc.), AD dementia patients revealed the worst cognitive function than CN and MCI subjects. For the imaging endophenotypes, AD group had the most smallest cortical volume of hippocampus, middle temporal and entorhinal cortex as compared to MCI and NC group with MRI method (*P* < 0.01). Moreover, the lowest cerebral glucose metabolism rate and the highest Aβ tracer retention were found in AD patients than MCI and CN individuals.

**Table 1:** The characteristics of the ADNI subjects at baseline

Characteristics	CN		MCI		AD		*P*^a^
Age (years)	281	74.51±5.56	483	72.28±7.45	48	75.51±9.23	-
Gender (male/female)	281	136/145	483	282/201	48	30/18	-
Education (years)	281	16.41±2.66	483	15.98±2.82	48	15.73±2.62	0.08
APOE ε4 (0/1/2)	281	204/70/7	483	262/180/41	48	14/25/9	<0.01
CDR-SB	207	0.03±0.13	406	1.44±0.87	47	4.44±1.69	<0.01
MMSE	281	29.07±1.15	483	27.89±1.69	48	22.96±2.03	<0.01
ADAS-cog	281	9.06±4.23	480	15.30±6.65	48	29.80±8.44	<0.01
RAVLT	280	44.83±9.60	483	36.16±10.86	47	22.32±7.84	<0.01
FAQ	281	0.17±0.66	481	2.85±3.99	48	12.6±7.14	<0.01
Hippocampus (mm3)	257	7344±895	422	6996±1126	39	5757±948	<0.01
Middle Temporal (mm3)	257	20298±2600	422	20186±2735	39	17776±3230	<0.01
Entorhinal (mm3)	257	3803±650	422	3610±723	39	2919±705	<0.01
CMRgl	207	6.55±0.55	406	6.32±0.64	47	5.30±0.72	<0.01
SUVR	152	1.12±0.19	323	1.20±0.22	46	1.39±0.22	<0.01

CN, cognitively normal; MCI, mild cognition impairment; AD, Alzheimer's disease; CDRSB, Clinical Dementia Rating scale sum of boxes; ADAS, Alzheimer's disease Assessment Scale; MMSE, Mini-Mental State Exam; RAVLT, Rey Auditory Verbal Learning Test; FAQ, Functional Activities Questionnaire; FDG, Cerebral Glucose Metabolism Rate measured with fluorodeoxyglucose-positron emission tomography(FDG-PET).

*P*^a^ values for continuous variables are from one-way analysis of variance (ANOVA). P values for categorical data are from chi square test. Data are given as mean ± standard deviation unless otherwise indicated

### Brain structure and *MS4A6A* genotypes

**Figure 1:**
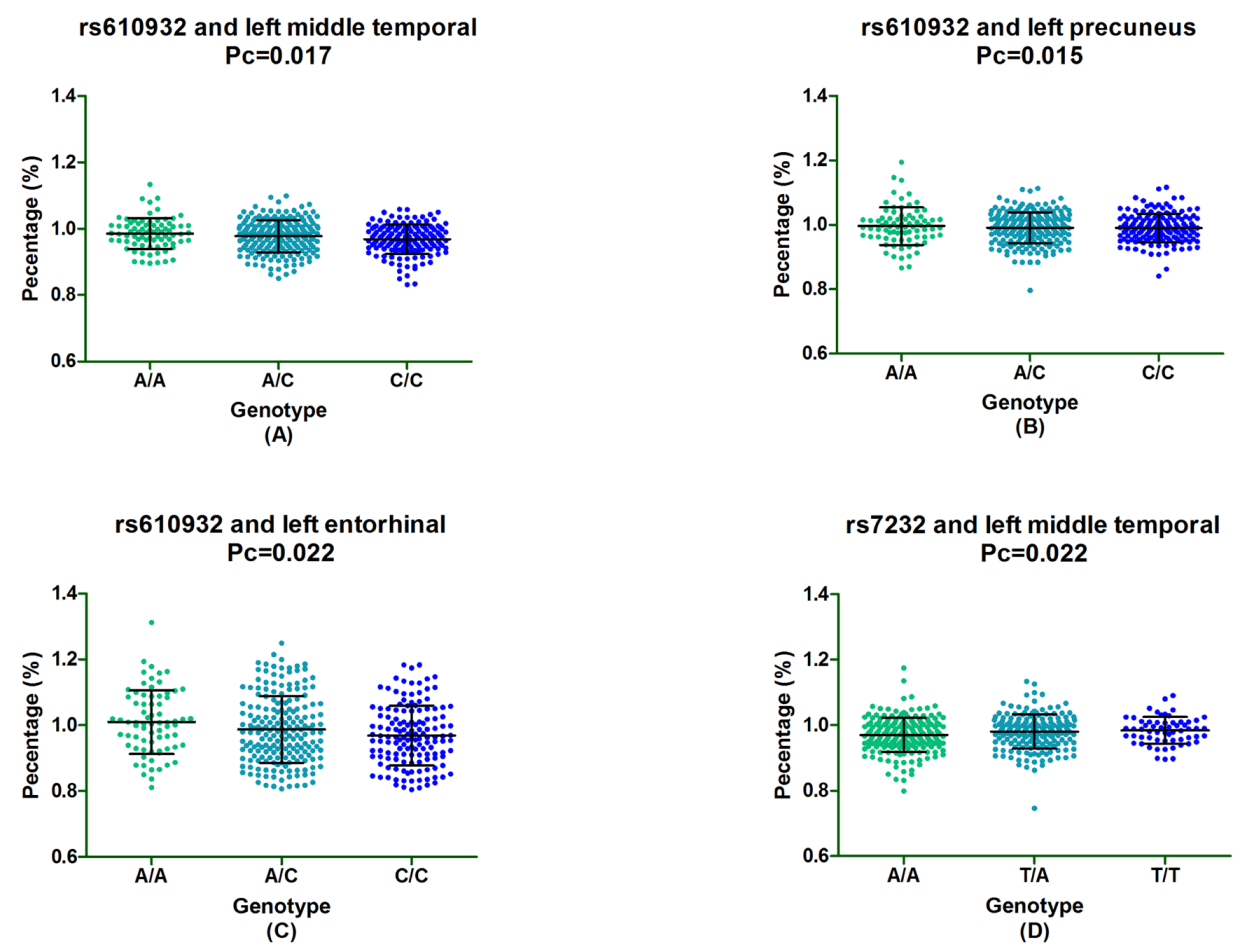
The significant correlation between *MS4A6A* loci and morphological changes of AD specific structure on MRI in all group analysis Figure 2A-2C depicted that rs610932 was associated with the atrophy rate of left middle temporal, left precuneus and left entorhinal in one year follow-up study. Figure 2D depicted that rs7232 was associated with the atrophy rate of left middle temporal after one-year follow-up.

In the follow-up study of one-year, we found that rs610932 were significantly associated with the percentage of decreases in the volume of left middle temporal, left precuneus and left entorhinal and these significant associations still survived after the FDR correction (*Pc* = 0.017, *Pc* = 0.015 and *Pc* = 0.022, respectively). In addition, rs610932 with minor A allele carriers decreased the atrophy rate of left middle temporal, left precuneus and left entorhinal in a dose-dependent manner (AA>CA>CC) (Figure 1A-1C). Moreover, the locus variation at rs7232 also significantly correlated the atrophy rate of left middle temporal (*Pc* = 0.022) and minor T allele carriers had less loss in the volume of left middle temporal than A allele homozygotes subjects (TT>TA>AA) (Figure 1D). SNPs also showed significant association with changes of volume in multiple ROIs, including left posterior cingulate (rs610932), left precuneus (rs610932), left parahippocampal (rs7232) and left entorhinal (rs7232) after two-year follow-up, but all associations failed to survive after the FDR correction. In our all group study, there was no evidence for an effect of rs12453 on structural MRI in these above ROIs (Supplementary Table 2).

**Figure 2:**
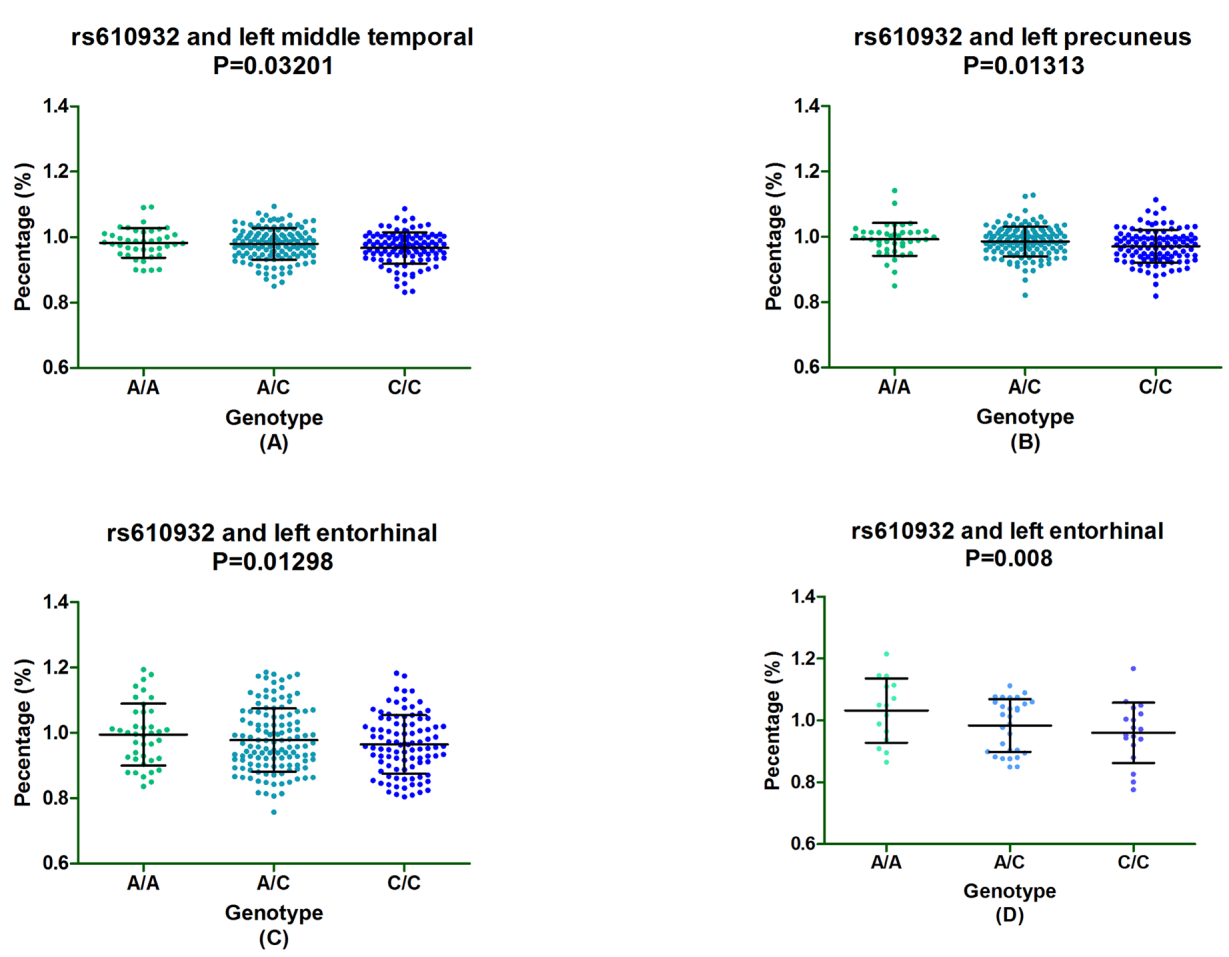
The significant correlation between rs610932 and morphological changes of AD specific structure on MRI in subgroup analysis after one-year follow-up Figure 2A-2C depicted that rs610932 was associated with the atrophy rate of left middle temporal, left entorhinal and left precuneus in MCI group. Figure 4D depicted that rs610932 was associated with the atrophy rate of left entorhinal in NC group.

**Figure 3:**
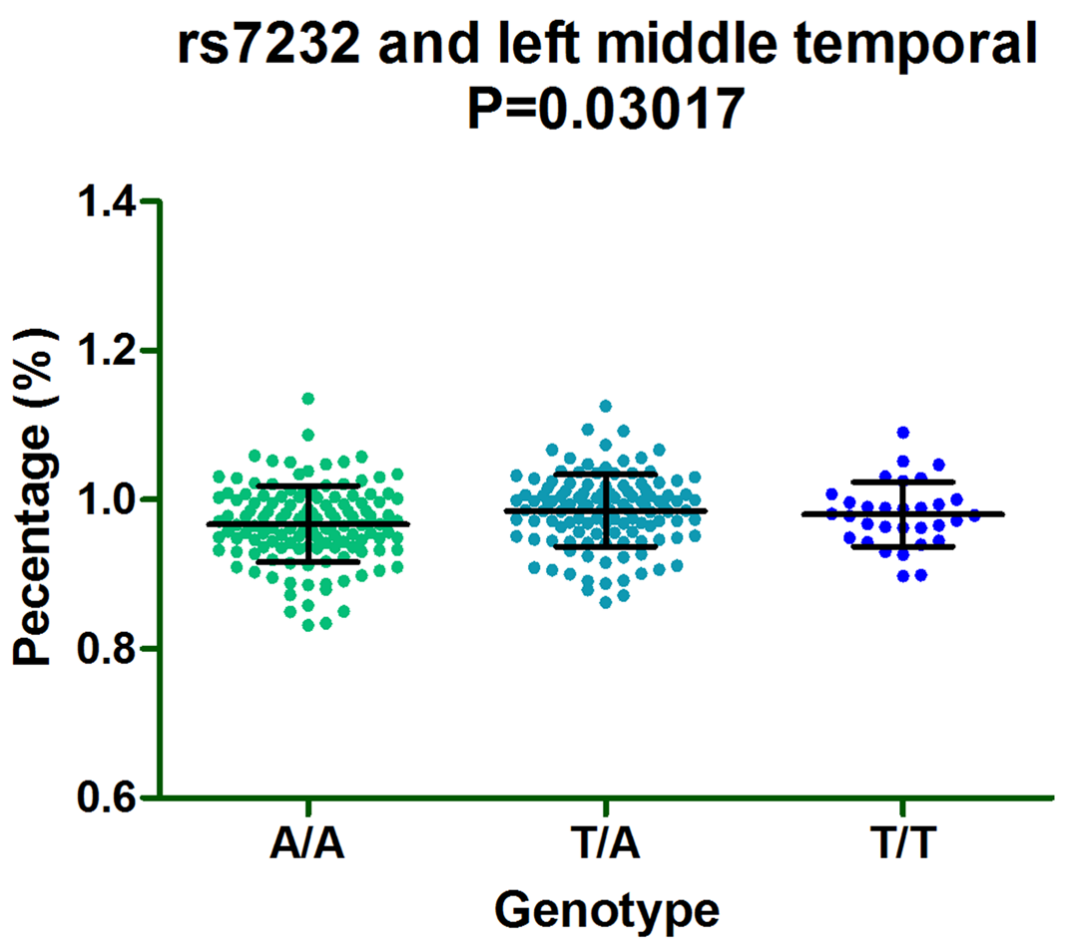
The significant correlation between rs7232 and morphological changes of AD specific structure on MRI in MCI group after one-year follow-up

Furthermore, subgroup analysis discovered that rs610932 significantly correlated the left middle temporal, precuneus and entorhinal of MCI individuals (*P* = 0.03201, *P* = 0.01313 and *P* = 0.01298, respectively) (Figure 2A-2C) and also altered the left entorhinal volume (*P* = 0.007726) in CN subgroup in the follow-up of one year (Figure 2D). Moreover, rs7232 effected the atrophy rate of left middle temporal only in MCI group (*P* = 0.03017) in one year follow-up study (Figure 3).

Rs610932 has been validated to be linked to AD in the many large GWAS, we also found that rs610932 and rs7232 were verified to associate with AD (*P* = 3.07×10^-9^ and *P* = 1.73×10^-10^, respectively) in meta-analysis of 74 046 participants.

### Association of *MS4A6A* with cerebral glucose metabolism

Taken amygdala, posterior cingulate and temporal cortex as ROIs, we analyzed the influence of *MS4A6A* genotypes on cerebral metabolism rate of glucose (CMRgl) on FDG-PET imaging. Unfortunately, no significant association of three SNPs (rs610932, rs7232 and rs12453) with the CMRgl on FDG-PET was found in the follow up study (Supplementary Table 3).

### Association of *MS4A6A* and Aβ deposition

Regarding the analysis of AV45-PET in frontal, parietal and temporal cortex and cingulate, we also not found the association of *MS4A6A* with Aβ deposition in the follow-up study (Supplementary Table 3).

## DISCUSSION

Our study demonstrated that *MS4*A6A genotypes correlated the brain atrophic rate of some special brain regions, but not associated with cerebral glucose metabolism and Aβ deposition in the follow-up study. MS4A6A polymorphisms (rs610932 and rs7232) changed the atrophy rate of some brain structural in a dose-dependent manner. Rs610932 with minor A allele carriers decreased the atrophy rate of left middle temporal, left precuneus and left entorhinal and rs7232 with minor T allele carriers had less loss in the volume of left middle temporal than A allele homozygotes subjects. In all group analysis, we found rs610932 were decreased in the percentage of volume loss of left middle temporal, left entorhinal and left precuneus in a dose-dependent manner (AA>CA>CC). Moreover, rs7232 mainly correlated with atrophy rate of left middle temporal and minor T allele carriers had less loss in the volume of left middle temporal than A allele homozygotes subjects (TT>TA>AA). But no association was found in rs12453 with any above ROIs. In order to identify that at which stage these variants impacted these pathological markers in the pathogenesis of AD, the significant associations between these positive MS4A6A loci and suggestive phenotypes in total group will be further detected in subgroup (CN, MCI and AD). Subgroup analysis found those brain structures (left entorhinal, left middle temporal and left precuneus) atrophy rate were significantly correlated with MS4A6A variants.

Two large GWAS had identified the minor T allele of rs610932 as a protective allele in Europeans, and this result had been successfully validated by our group in Han Chinese population. It is well-known that the sequence and structural motifs of 3′-UTR regulated the process of messenger RNA stability, translation, and localization. The SNP rs610932 which located in the 3′-UTR of the MS4A6A gene may participate in the gene expression process and regulates the expression of the MS4A6A [34]. Previous studies have shown that rs610932 and rs7232 were strongly associated with the levels of MS4A6A expression in blood from MCI and AD patients (*p* < 0.05) [35]. Besides, rs610932, rs7232 and rs12453 were found to nominally significant in relation to MS4A6A expression in cerebellum and temporal cortex while increased minor T allele copies of rs610932 are accompanied with lower MS4A6A gene expression [15, 35]. Mounting evidences have suggested that high levels of MS4A6A are increased prevalence risk of AD and in relation to Braak tangle and Braak plaque scores [25-27]. Moreover, Martiskainen and his colleagues reported that highly MS4A6A expression was significantly correlated to AD-related neurofibrillary pathology and tau phosphorylation in the postmortem inferior temporal cortex in a sample containing 60 participants with different levels of AD-related neurofibrillary pathology [28]. Although the exact mechanisms underlying the effects of MS4A6A on the occurrence and development of AD are still largely unknown, several members of MS4A cluster are found to participate in the regulation of calcium signaling [36-40] and immune-system function [41-43]. All these above evidence, along with our finding, it could be linked to a hypothesis that the role of *MS4A6A* variants in AD might be mediated by modifying neurodegenerative changes, possibly by affecting calcium signaling and immune-system function in these regions and subsequently influencing memory function.

Endophenotype-based design would gain greater power and smaller sample size requirements to detect disease susceptibility loci than traditional case-control designs. Although lots of researches have identified the effect of genotype on AD-related neuroimaging phenotypes, to our knowledge, our investigation was the first study to explore the role of *MS4A6A* genotypes in neuroimaging phenotypes and detected that *MS4A6A* variants was significantly associated with the percentage change of volume of middle temporal, entorhinal and precuneus. But there are several potential limitations in this study. Firstly, the ADNI sample size was relatively small when considering follow-up imaging data of specific genotypes, and more studies are needed from a larger dataset to evaluate the association between MS4A6A and AD-related neuroimaging phenotypes. Secondly, only left hemisphere structures are associated with genetic variants, but whether the subjects are all right-handed or not is unknown. Thirdly, two years follow-up may be too short to observe the remark effect of *MS4A6A* on the changes of structural volumetric MRI, and an longer years of follow-up is need to replicate these findings. Finally, although GWAS identified several variants within *MS4A6A* (such as rs662196, rs583791 and rs983392), but we cannot extracted these SNPs from the GWAS of ADNI for all included participants [16, 35, 44]. So, more association studies with larger number of subjects and longer follow-up are still eager to sustain the present research findings.

In conclusion, our study provides evidence that supporting the possible role of *MS4A6A* genotypes in influencing AD-related neuroimaging phenotypes. These findings further confirmed the hypothesis that *MS4A6A* genetic variants may modulate the alteration of the biomarkers of neuronal degeneration or injury and then influence the risk of AD. Nonetheless, further work is essential to explain the effect of *MS4S6A* on AD in a larger sample with longer follow-up.

## MATERIALS AND METHODS

### ADNI dataset

The ADNI dataset is a consortium of the National Institute on Aging, the National Institute of Biomedical Imaging and Bioengineering, the Food and Drug Administration, private pharmaceutical companies, and nonprofit organizations to develop serial MRI, positron emission tomography (PET), other biological procedures, and neuropsychological and clinical assessment in cognitively normal older subjects, early or late MCI subjects, and early AD subjects (http://www.adni-info.org). The primary purpose of ADNI is to establish an accessible database that describes longitudinal changes in brain structure, function and metabolism according to parallel clinical, cognitive, and biochemical data. To date, the three protocols have recruited over 1,500 adults to participate in the research, ages from 55 to 90 [45]. Informed consent was acquired from all subjects or from authorized representatives and with approval from the institutional review boards of all participating centers.

### Subjects

The subjects who classified as normal controls, MCI subjects, or AD subjects used in this research were downloaded from the ADNI database. The subjects with AD between the ages of 55-90, with an Mini-Mental State Examination (MMSE) score of 20-26 inclusive and meeting the NINCDS/ADRDA criteria for probable AD, and having a Clinical Dementia Rating (CDR) of 0.5 or 1. While the subjects with MCI have memory complaints, fulfilled the MMSE score between 24 and 30 and the CDR score was 0.5, essentially preserved activities of daily living. On the other hand, all individuals included in this study were free from any serious neurological disease except for possible AD, current or past history of brain lesions or head trauma, or psychoactive medication use [45]. Other detailed specification can be found on the ADNI cohort online (http://adni.loni.usc.edu/).

### SNPs selection

In our study, MS4A6A genotypes data were downloaded from the ADNI GWAS using PLINK format. The quality control (QC) procedures were performed using PLINK software, and meeting the following criteria for the SNPs will be included in our research: Hardy-Weinberg equilibrium test P >0.001, minimum call rates >90%, minimum minor allele frequencies (MAF) >0.01.

We first selected four *MS4A6A* loci (rs610932, rs583791, rs662196 and rs138650483) which have been reported to be significantly correlated with AD in published GWAS for analysis [16, 46, 47]. In addition, we further searched seven promising candidate loci (rs7232, rs12453, rs646924, rs632185, rs983392, rs17602572 and rs2278867) from meta-analysis and replication studies [15, 17, 18, 44, 48, 49] (Supplementary Table 1). Although 11 SNPs were identified in the initial screening, 8 SNPs were further excluded due to their absence in ADNI. Therefore, we selected 3 loci (rs610932, rs7232 and rs12453), which meeting inclusion criteria for the SNP quality control, to research the association between MS4A6A genotypes and brain structure (Table 2).

**Table 2:** Characteristics of three SNPs included for our analysis

SNP	Chr	Position	Minor allele	SNP source	MAF	H-W (p value)	Ref.
rs610932	11	3′-untranslated region (3′-UTR)	A	GWAS & Meta-analysis & Replication	0.406	0.9325	[15-18],[30]
rs7232	11	Nonsynonymous	T	Replication	0.348	0.2644	[20],[33]
rs12453	11	Synonymous	C	Replication	0.373	0.7207	[33]

**Abbreviation:** MAF= Minor Allele Frequency; SNP= Single Nucleotide Polymorphism; H-W= Hardy-Weinberg

### Brain structures on MRI

All data of structural volumetric MRI were downloaded from the ADNI dataset for free. This technology provided by the University of California, San Francisco (UCSF) medical center. A detailed description of imaging data acquisition and processing can be acquired in other papers [50]. Previous studies have demonstrated that brain atrophy may begin to emerge many years before the clinical symptoms of mild MCI that associated to evidences of AD. We defined six cerebral areas, including hippocampus, parahippocampal, entorhinal, middle temporal, posterior cingulate and precuneus, as regions of interest as they are known to be affected by AD and mounting researches have been previously validated their atrophy in AD *via* MRI studies. In this study we calculated one and two years percent volumetric changes of the most discriminant ROIs for longitudinal analysis to analyze their associations with MS4A6A genotypes (Supplementary Table 2).

### Glucose metabolism on imaging

The cerebral metabolic rate for glucose (CMRgl) on FDG-PET analysis data were provided by both UC Berkeley and Lawrence Berkeley National Laboratory [51]. We extracted the data from the site (http://adni.loni.usc.edu/data-samples/access-data/). Their researchers used five brain regions (right and left right temporal gyrus, right and left angular gyrus and bilateral posterior cingulate) as ROIs to analysis (Supplementary Table 3). We downloaded FDG-PET analysis data from LONI (http://loni.usc.edu/), and subsequently these images were spatially normalized in Statistical Parametric Mapping (SPM) to the Montreal Neurological Institute (MNI) PET template. At last, the mean counts of these five ROIs for each subject's FDG scans at each time point were used to compute the intensity values with SPM subroutines [52].

### Aβ deposition on AV45-PET imaging

UC Berkeley-AV45 analysis dataset offered the PET imaging data with amyloid tracer, florbetapir (AV-45) at site (http://adni.loni.usc.edu/data-samples/access-data/). On this site, we can found the detailed description of PET image acquisition and processing. A native-space MRI scan for each subject was segmented with Freesurfer (version 4.5.0) to define cortical grey matter ROIs, including frontal, lateral parietal, lateral temporal anterior/posterior cingulate, which make up a summary cortical ROIs [53, 54] (Supplementary Table 3). The institute used cerebellum ROIs (grey matter only) as a reference region. Firstly, each florbetapir scan was applied to the corresponding MRI, and then they calculated the mean florbetapir uptake within the cortical and reference region. Finally, florbetapir standard uptake value ratios (SUVRs) were generated by averaging across the four ROIs and dividing this average by whole cerebellum.

### Statistical analysis

Demographic and clinical characteristics of subjects (AD, MCI and control subjects) were described either in terms of means and standard deviations (SD) if quantitative or in terms of proportions. One-way analysis of variance (ANOVA) was used to compare the differences in continuous variables and chi-square test was used to examine the categorical data. Furthermore, taken age, gender, education and number of APOE ε4 allele as covariates, the multiple linear regression model was used to evaluate the possible associations between various phenotypes and MS4A6A genotypes. At first, we computed the influences of *MS4A6A* loci on the percentage change of the imaging phenotypes in follow-up study. Secondly, the significant associations between these positive MS4A6A loci and suggestive phenotypes in total group will be further detect in subgroup (CN, MCI and AD subjects) to distinguish at which stage these variants impacted these pathological markers in the pathogenesis of AD. Finally, we verify the associations between these positive loci in this study and AD patients from a large database which included 74 046 individuals of European descent [44].

Statistical analyses were performed using R 3.12. According to the method invented by Hochberg and Benjamini for the number of tests, we performed the false discovery rate (FDR) to account for multiple testing [55]. All tests were two-sided and FDR-corrected *P* < 0.05 was considered statistically significant.

## SUPPLEMENTARY MATERIALS TABLES


