# Supplementary material for: MS4A6A genotypes are associated with the atrophy rates of Alzheimer's disease related brain structures
Source: Oncotarget. 2016 May 23;7(37):58779–88. doi: 10.18632/oncotarget.9563 (PMC5312275; doi:10.18632/oncotarget.9563)
Supplement: Supplementary file 1 [file oncotarget-07-58779-s001.pdf]

MS4A6A genotypes are associated with the atrophy rates of Alzheimer's disease related brain structures

Supplementary Materials

Supplementary table 1- Information of SNP in the process of SNP selection

SNP	Chr	Position	P Value	Population type	SNP source	Inclusion	Ref.
rs610932	11	3'UTR	1.4×10^{-6}	Europe and the USA	GWAS	yes	[19734902]
rs610932	11	3'UTR	1.2×10^{-16}	GERAD1&2, EADI1&2, ADNI, TGEN1, Decode, AD-IG, CHARGE, Mayo2 and ADGC	Meta-analysis	yes	[21460840]
rs610932	11	3'UTR	<0.001	Chinese Han population	Replication	yes	[22382309]
rs610932	11	3'UTR	0.019	Northern Han Chinese population	Replication	yes	[23232270]
rs610932	11	3'UTR	0.043	the cross-European AddNeuroMed or the King's Health Partners/Maudsley BRC and Dementia Case Registry at King's Health Partners studies	Replication	yes	[24064185]
rs7232	11	Coding, non-	0.045	the cross-European AddNeuroMed or the King's	Replication	yes	[24064185]

		synonymousc		Health Partners/Maudsley BRC and Dementia Case Registry at King's Health Partners studies			
rs7232	11	Nonsynonymous	0.1130	Korean Population	Replication	yes	[22975751]
rs12453	11	Synonymous	0.0831	Korean Population	Replication	yes	[22975751]
rs662196	11	Intronic	5.2×10^{-6}	Europe and the USA	GWAS	no	[19734902]
rs583791	11	Intronic	5.3×10^{-6}	Europe and the USA	GWAS	no	[19734902]
rs583791	11	Intronic	1.868×10^{-9}	Spanish population and four other public GWASs （ADNI， GenADA， NIA and TGEN）	Meta-analysis	no	[21627779]
rs583791	11	Coding, non-synonymousc	0.033	the cross-European AddNeuroMed or the King's Health Partners/Maudsley BRC and Dementia Case Registry at King's Health Partners studies	Replication	no	[24064185]
rs17602572	11	NA	1.151×10^{-5}	Spanish population and four other public GWASs （ADNI， GenADA， NIA and TGEN）	Meta-analysis	no	[21627779]
rs2278867	11	NA	9.928×10^{-6}	Spanish population and four other public GWASs （ADNI， GenADA， NIA and TGEN）	Meta-analysis	no	[21627779]
rs646924	11	Intron	0.4876	Korean Population	Replication	no	[22975751]
rs632185	11	Upstream	0.5101	Korean Population	Replication	no	[22975751]

rs983392	11	NA	6.1×10^{-16}	ADGC, CHARGE, EADI, GERAD and Austria, Belgium, Finland, Germany, Greece, Hungary, Italy, Spain, Sweden, UK and United States	Meta-analysis	no	[24162737]
rs138650483	11	exonic/splicing	NA	Caribbean Hispanics families, NIA-LOAD Family Study and Canadian individuals of European ancestry	GWAS	no	[26101835]

Abbreviation: 3'UTR =3′-untranslated region, ADGC=Alzheimer's Disease Genetics Consortium, AD-IG=German Alzheimer's Disease Integrated Genome Research Network, ADNI= Alzheimer's Disease Neuroimaging Initiative cohort, CHARGE =Cohorts for Heart and Aging Research in Genomic Epidemiology, EADI=European AD Initiative Consortium, GenADA=Genotype-Phenotype Associations in Alzheimer's Disease Study, GERAD=Genetic and Environmental Risk in Alzheimer's Disease Consortium, GWAS=genome-wide association study, NIA-LOAD=National Institute on Aging Late-Onset Alzheimer's Disease cohort, SNP=single nucleotide polymorphism, TGEN=Translational Genomics Research Institute cohort.

Supplementary table 2- Detailed results of the associations of *MS4A6A* loci with brain regions on MRI in all group

ROI	SNP	Follow-up (1 year)			Follow-up (2 year)		
		Sample (L/R)	P-value (L/R)	P-FDR (L/R)	Sample (L/R)	P-value (L/R)	P-FDR (L/R)
Middle Temporal (volume)	rs610932	442/442	0.005532/0.6863	0.0166/0.84	233/233	0.4559/0.2453	0.6128/0.3679
	rs7232	442/442	0.01443/0.6805	0.02165/0.84	233/233	0.1734/0.1203	0.5201/0.3609
	rs12453	442/442	0.06276/0.84	0.06276/0.84	233/233	0.6128/0.4107	0.6128/0.4107
Entorhinal (volume)	rs610932	442/442	0.007245/0.9723	0.02174/0.9723	233/233	0.104/0.3374	0.104/0.6116
	rs7232	442/442	0.05276/0.7817	0.5276/0.9723	233/233	0.03268/0.4773	0.0757/0.6116
	rs12453	442/442	0.05114/0.8898	0.05276/0.9723	233/233	0.05046/0.6116	0.0757/0.6116
Parahippocampal (volume)	rs610932	442/442	0.3798/0.2431	0.09627/0.5848	232/233	0.1446/0.2052	0.1446/0.4078
	rs7232	442/442	0.9627/0.5848	0.09627/0.5848	232/233	0.02415/0.3147	0.07244/0.4078
	rs12453	442/442	0.959/0.3933	0.09627/0.5848	232/233	0.09594/0.4078	0.1439/0.4078
Posterior Cingulate	rs610932	442/442	0.4828/0.1853	0.9221/0.556	233/233	0.02287/0.0858	0.06862/0.2142

**(volume)**	**rs7232**	**442/442**	**0.9221/0.7442**	**0.9221/0.7442**	**233/233**	**0.422/0.2321**	**0.422/0.2321**
	**rs12453**	**442/442**	**0.7418/0.3937**	**0.9221/0.5906**	**233/233**	**0.2189/0.1428**	**0.3284/0.2142**
**Precuneus (volume)**	**rs610932**	**442/442**	**0.004913/0.1585**	**0.01474/0.4693**	**233/233**	**0.0265/0.8309**	**0.7949/0.89**
	**rs7232**	**442/442**	**0.06572/0.4095**	**0.06572/0.4693**	**233/233**	**0.1248/0.89**	**0.1248/0.89**
	**rs12453**	**442/442**	**0.5204/0.4693**	**0.06572/0.4693**	**233/233**	**0.08419/0.7877**	**0.1248/0.89**
**Hippocampus (volume)**	**rs610932**	**442/442**	**0.1491/0.8136**	**0.3366/0.8136**	**233/233**	**0.2056/0.6141**	**0.3304/0.9842**
	**rs7232**	**442/442**	**0.3771/0.5116**	**0.3771/0.7675**	**233/233**	**0.3304/0.8039**	**0.3304/0.9842**
	**rs12453**	**442/442**	**0.2244/0.3082**	**0.3366/0.7675**	**233/233**	**0.2272/0.9842**	**0.3304/0.9842**
**CA1 region (volume)**	**rs610932**	**442/442**	**0.7154/0.3326**	**0.8769/0.3871**	**232/232**	**0.7999/0.9491**	**0.8342/0.9491**
	**rs7232**	**442/442**	**0.2333/0.1464**	**0.6998/0.3871**	**232/232**	**0.4937/0.1418**	**0.8342/0.3288**
	**rs12453**	**442/442**	**0.8769/0.3871**	**0.8769/0.3871**	**232/232**	**0.8342/0.2192**	**0.8342/0.3288**
**Entorhinal (thickness)**	**rs610932**	**442/442**	**0.9107/0.3884**	**0.91070.4859**	**233/233**	**0.8667/0.5608**	**0.8667/0.9687**
	**rs7232**	**442/442**	**0.8557/0.4859**	**0.9107/0.4859**	**233/233**	**0.2491/0.8301**	**0.7473/0.9687**
	**rs12453**	**442/442**	**0.6601/0.4552**	**0.9107/0.4859**	**233/233**	**0.5125/0.9687**	**0.7688/0.9687**

Abbreviation: ROI=regions of interest; SNP=single nucleotide polymorphism; L=Left; R=Right.

Supplementary table 3- Detailed results of the associations of *MS4A6A* loci with Glucose metabolism or Aβ deposition on imaging in all group

ROI	SNP	Follow-up (2 year)		
		Sample (L/R)	P-value (L/R)	P-FDR (L/R)
Angular (CMRg)	rs610932	299/299	0.6556/0.6735	0.6556/0.6735
	rs7232	299/299	0.3834/0.2897	0.64/0.4605
	rs12453	299/299	0.4266/0.307	0.64/0.4605
Post Cigulum (B) (CMRg)	rs610932	299	0.7415	0.7415
	rs7232	299	0.1015	0.4054
	rs12453	299	0.2703	0.4054
Temporal (CMRg)	rs610932	299/299	0.4318/0.9304	0.4318/0.9304
	rs7232	299/299	0.1015/0.4946	0.2707/0.7419
	rs12453	299/299	0.1804/0.4076	0.2707/0.7419

Cingulate (AV45)	rs610932	381	0.8351	0.866
	rs7232	381	0.9728	0.866
	rs12453	381	0.7047	0.866
Frontal (AV45)	rs610932	381	0.7338	0.9431
	rs7232	381	0.9413	0.866
	rs12453	381	0.581	0.866
Parietal (AV45)	rs610932	381	0.6038	0.6322
	rs7232	381	0.8355	0.3966
	rs12453	381	0.4472	0.3966
Temporal (AV45)	rs610932	381	0.4286	0.6505
	rs7232	381	0.588	0.5382
	rs12453	381	0.3207	0.5382
